# Global perspectives of ophthalmologists on artificial intelligence adoption in clinical practice

**DOI:** 10.1186/s40942-025-00764-4

**Published:** 2025-11-25

**Authors:** Lorenzo Ferro Desideri, Yousif Subhi, Janice Roth, Enrico Bernardi, Gustavo Barreto Melo, Edmund Tsui, Adrian T. Fung, Nicola Sagurski, Charles Wykoff, Hasenin Al-Khersan, Jose Carlo M. Artiaga, Ogugua Okonkwo, Hung-Da Chou, Pierre-Henry Gabrielle, Jay Chhablani, Martin Zinkernagel, Rodrigo Anguita

**Affiliations:** 1 Department of Ophthalmology, Inselspital, Bern University Hospital, University of Bern, Freiburgstrasse 15, Bern, CH-3010 Switzerland; 2 Department for BioMedical Research, University of Bern, Murtenstrasse 24, Bern, CH-3008 Switzerland; 3 Bern Photographic Reading Center, Inselspital, Bern University Hospital, University of Bern, Bern, Switzerland; 4 Department of Ophthalmology, Rigshospitalet, Glostrup, Denmark; 5 Department of Clinical Medicine, University of Copenhagen, Copenhagen, Denmark; 6 Department of Clinical Research, University of Southern Denmark, Odense, Denmark; 7 Department of Ophthalmology and Visual Sciences, Federal University of Sao Paulo, Sao Paulo, Brazil; 8 Sergipe Eye Hospital (Hospital de Olhos de Sergipe), Aracaju, Sergipe Brazil; 9 Stein Eye Institute, David Geffen School of Medicine at UCLA, Los Angeles, CA USA; 10 Faculty of Medicine and Health, The University of Sydney, Sydney, NSW Australia; 11 Department of Ophthalmology, Westmead Hospital, Sydney, NSW Australia; 12 Department of Ophthalmology, Faculty of Medicine and Health Sciences, Macquarie University, Sydney, Australia; 13 Zhongshan Ophthalmic Centre, Sun Yat-sen University, Guangzhou, China; 14 Retina Consultants of Texas, Retina Consultants of America, Houston, TX USA; 15 Department of Clinical Epidemiology, College of Medicine, University of the Philippines Manila, Manila City, Philippines; 16 Department of Ophthalmology, Eye Foundation Hospital and Eye Foundation Retinal Institute, Lagos, Lagos State Nigeria; 17 Department of Ophthalmology, Chang Gung Memorial Hospital, Linkou Medical Center, Taiwan and College of Medicine, Chang Gung University, Taoyuan, Taiwan; 18 Ophthalmology Department, Dijon University Hospital, Dijon, France; 19 Ophthalmology, University of Pittsburgh, Pittsburgh, PA USA; 20 Moorfields Eye Hospital NHS Foundation Trust, London, UK

**Keywords:** Artificial intelligence, AI in ophthalmology, AI training, Ethical concerns

## Abstract

**Background:**

Artificial intelligence (AI) is rapidly expanding in ophthalmology, yet its adoption in daily practice remains limited. Understanding clinicians’ perspectives is essential to address barriers and guide targeted education.

**Methods:**

We conducted a cross-sectional international survey of licensed ophthalmologists from 45 countries across all continents between October 2024 and February 2025. The questionnaire evaluated AI familiarity and use as primary outcomes, as well as perceived clinical impact, ethical concerns, and training preferences of participants. Descriptive and comparative analyses were conducted across world region, practice type, and professional seniority.

**Results:**

A total of 622 ophthalmologists completed the survey. While 69.5% anticipated a moderate-to-very potential for AI to improve clinical outcomes, only 7.2% reported regular use. Familiarity with AI was significantly higher among academic clinicians (*p* = 0.0011), whereas 49.6% reported no knowledge of specific AI tools. Key barriers included lack of training (20.5%), implementation costs (16.5%), and reliability concerns (12.9%). Ethical issues most frequently cited were algorithmic bias (44.2%), liability (36.7%), and reduced physician–patient interaction (19.9%). Ophthalmologists with > 20 years of experience were more likely to support AI adoption (OR 1.5). Interest in AI education was high (75.1%), with a preference for online and structured formats and calls for earlier integration into medical curricula.

**Conclusions:**

Despite broad recognition of AI’s potential in ophthalmology, adoption remains low and familiarity limited. Lack of training, cost, and ethical concerns represent key barriers. Tailored, accessible education and institutional support are urgently needed to facilitate safe and effective AI integration into clinical practice.

## Background

Artificial intelligence (AI) is rapidly transforming modern medicine with ophthalmology currently representing one of the lead specialties impacted by AI-driven innovation [1]. AI applications in ophthalmological diseases have demonstrated notable potential in clinical decision support, disease diagnosis, and treatment monitoring [2]. From detecting diabetic retinopathy and differentiating optic neuropathies using fundus photography to estimating glaucoma progression through visual field data, AI is increasingly being integrated into ophthalmic practice [3–5].

AI in ophthalmology relies on machine learning, deep learning, natural language processing, and computer vision. More recently, the emergence of large language models (LLMs) has further expanded the potential applications of AI in medicine and ophthalmology, offering possible support in clinical decision, automated report generation, patient communication, and medical education [6, 7]. AI applications have been utilized for ophthalmological triage process, refining diagnostic and prognostic accuracy for eye diseases, and have served as promising educational tool for both clinicians and patients [8–15]. As AI technologies continue to evolve, their adoption in clinical workflows is expected to increase, necessitating new approaches to education and training for eye care professionals [16].

Despite the growing presence of AI in ophthalmology, their successful implementation requires more than just technical advancements [7]. Ophthalmologists must develop a solid understanding of AI principles, its limitations, and the ethical considerations associated with AI-assisted decision-making. However, there is currently limited research evaluating ophthalmologists’ familiarity and experience with AI-driven tools, and their perspectives on how AI should be integrated into their training and continuing medical education [17].

We conducted a global survey of practicing ophthalmologists to assess AI familiarity, use, perceived clinical impact, and ethical concerns in routine care. The findings identify knowledge gaps and training priorities for safe, effective adoption.

## Methods

### Study design and participants

This study was designed as an international, cross-sectional survey to assess the perceptions, knowledge, concerns and expectations of ophthalmologists all over the world regarding the adoption of AI in clinical practice. The survey explored their perspectives on the need of education and training for the use of AI in ophthalmology.

The questionnaire was distributed online to ophthalmologists practicing in various geographic regions worldwide, aiming to capture diverse insights from professionals working in different healthcare settings. Participation was voluntary, and responses were anonymized. Eligible participants included licensed ophthalmologists of any subspecialty, working in academic institutions, private practices, or hospitals.

This survey study did not collect any personally identifiable information and was conducted anonymously among licensed ophthalmologists. Participation was voluntary and no incentives were provided. Informed consent was implied by survey completion. In accordance with the Declaration of Helsinki, formal ethical approval was not required.

### Ethical considerations

This survey complied with the principles of the Declaration of Helsinki. No personally identifiable data were collected, and participation was entirely voluntary and anonymous. Informed consent was implied by survey completion. The study protocol was reviewed by the local institutional review board, which confirmed that formal ethical approval was not required for anonymous survey-based research involving healthcare professionals.

### Outcomes

#### Primary outcome

Proportion of respondents reporting current use of AI-based tools in clinical practice (yes/no), overall and by region, years in practice, and AI familiarity.

#### Secondary outcomes

(i) Perceived impact of AI on clinical care and research; (ii) barriers to adoption (e.g., cost, workflow, validation, regulation); (iii) ethical–legal concerns (privacy, liability, bias); (iv) education and training needs (content, format, preferred resources); (v) future intent to adopt AI within 5–10 years; and (vi) regional differences across the above domains. Pre-specified subgroup analyses were conducted by geographic region, years in practice, and AI familiarity.

### Survey development

The survey was developed based on a review of existing literature on AI in medicine and ophthalmology, along with expert input from ophthalmologists and AI researchers. The questionnaire underwent internal validation through pilot testing with a small group of ophthalmologists (*n* = 10) to ensure clarity, relevance, and comprehensiveness. The final questionnaire was structured into seven sections:

1. **Demographics and background**: Country of practice, subspecialty, years in practice, and institutional setting.
2. **Knowledge and awareness of AI**: Familiarity with AI and its various subfields (e.g., machine learning, deep learning, LLMs and computer vision). Familiarity with AI referred to conceptual awareness (definitions, principles, example applications) and did not require prior or current hands-on use. General familiarity referred to conceptual awareness of AI and its applications in ophthalmology, whereas familiarity with specific subfields (e.g., ML, DL) reflected more technical knowledge.)
3. **Current adoption of AI in clinical practice**: Adoption of AI-based tools in daily practice and barriers to implementation.
4. **Perception of AI in research and clinical applications**: Views on the quality of AI research in ophthalmology and its practical impact.
5. **Future of AI in ophthalmology**: Expectations regarding AI’s role in ophthalmology over the next decade.
6. **Ethical and legal considerations**: Concerns regarding AI-driven clinical decision-making, data privacy, liability, and potential biases in patient care.
7. **AI in education and training**: Opinions on integrating AI into medical education and preferred learning resources.

Institution size was categorized a priori as small (1–5 practitioners), medium (6–20), large (21–50), and very large (> 50), according to predefined questionnaire options.

A full list of survey questions is provided in Table 1.

**Table 1:** Summary of survey questions administered to participants

Section	Topic	Question
1. Demographics and Background	Country	What country are you currently practicing in?
	Area of Practice	What is your primary area of practice?
	Years in Practice	How many years have you been practicing ophthalmology?
	Institution Size	What is the size of your institution or clinic?
	Academic Affiliation	Are you part of an academic or research institution?
2. Knowledge and Awareness of AI	AI Familiarity	How familiar are you with artificial intelligence (AI) in general?
	Awareness of AI Technologies	Which AI technologies are you aware of?
	Confidence in AI Understanding	Do you feel confident in your understanding of how AI algorithms are developed and validated?
3. Current Use of AI in Clinical Practice	AI Usage	Do you currently use any AI-based tools in your clinical practice?
	Types of AI Tools Used	Which types of AI-based tools have you used in your practice?
	Impact on Decision-Making	How has the use of AI tools impacted your clinical decision-making?
	Barriers to AI Use	What are the main barriers to using AI in your clinical practice?
4. Perception of AI in Research and Clinical Applications	Research Quality	How do you perceive the quality of current research on AI in ophthalmology?
	Translation into Practice	Do you believe that AI research in ophthalmology is translating into practical, usable tools?
5. Future of AI in Ophthalmology	Potential to Improve Outcomes	How do you rate the potential of AI to improve patient outcomes in ophthalmology?
	Beneficial Areas	What areas of ophthalmology do you believe will benefit the most from AI?
	Concerns about AI	What concerns do you have about the use of AI in ophthalmology?
	Future Role of AI	How do you imagine the role of AI in ophthalmology in the next 10 years?
	Willingness to Train	Would you be willing to undergo training to improve your understanding and use of AI in ophthalmology?
	Replacement of Ophthalmologists	Do you believe that AI will eventually replace human ophthalmologists in certain aspects of clinical practice?
6. Ethical and Legal Considerations	Steps for Responsible Use	What steps do you think should be taken to ensure the responsible use of AI in ophthalmology?
	Ethical Concerns	Are you concerned about the ethical implications of AI in patient care?
	Potential Bias	Do you think AI could lead to potential biases in patient care?
7. AI in Education and Training	Patient Confidentiality	How confident are you in AI’s ability to maintain patient confidentiality?
	Liability in Case of Errors	How should liability be handled if an AI tool contributes to a medical error?
	AI in Medical Education	Do you think AI should be integrated into the medical curriculum for future ophthalmologists?
	Learning Resources	What resources would you find most useful for learning about AI in ophthalmology?

### Survey administration

The questionnaire was distributed electronically via direct invitations to ophthalmologists worldwide. Data collection occurred between October 2024 and February 2025. Responses were collected using a private form, and participation was limited to one response per individual to prevent duplication. No incentives were provided for participation.

### Data analysis

Survey responses were analyzed using descriptive statistics, with categorical variables reported as frequencies and percentages. Subgroup analyses were performed based on geographic location, years in practice, and familiarity with AI. Associations between variables were assessed using chi-square tests or Fisher’s exact tests where appropriate. All statistical analyses were conducted using R software (version 4.2.0; R Foundation for Statistical Computing, Vienna, Austria). A regional subgroup analysis based on geographic location, grouping respondents into six regions: Africa, Asia, Europe, North America, South America, and Oceania.

## Results

### Participant characteristics

**Fig. 1:**
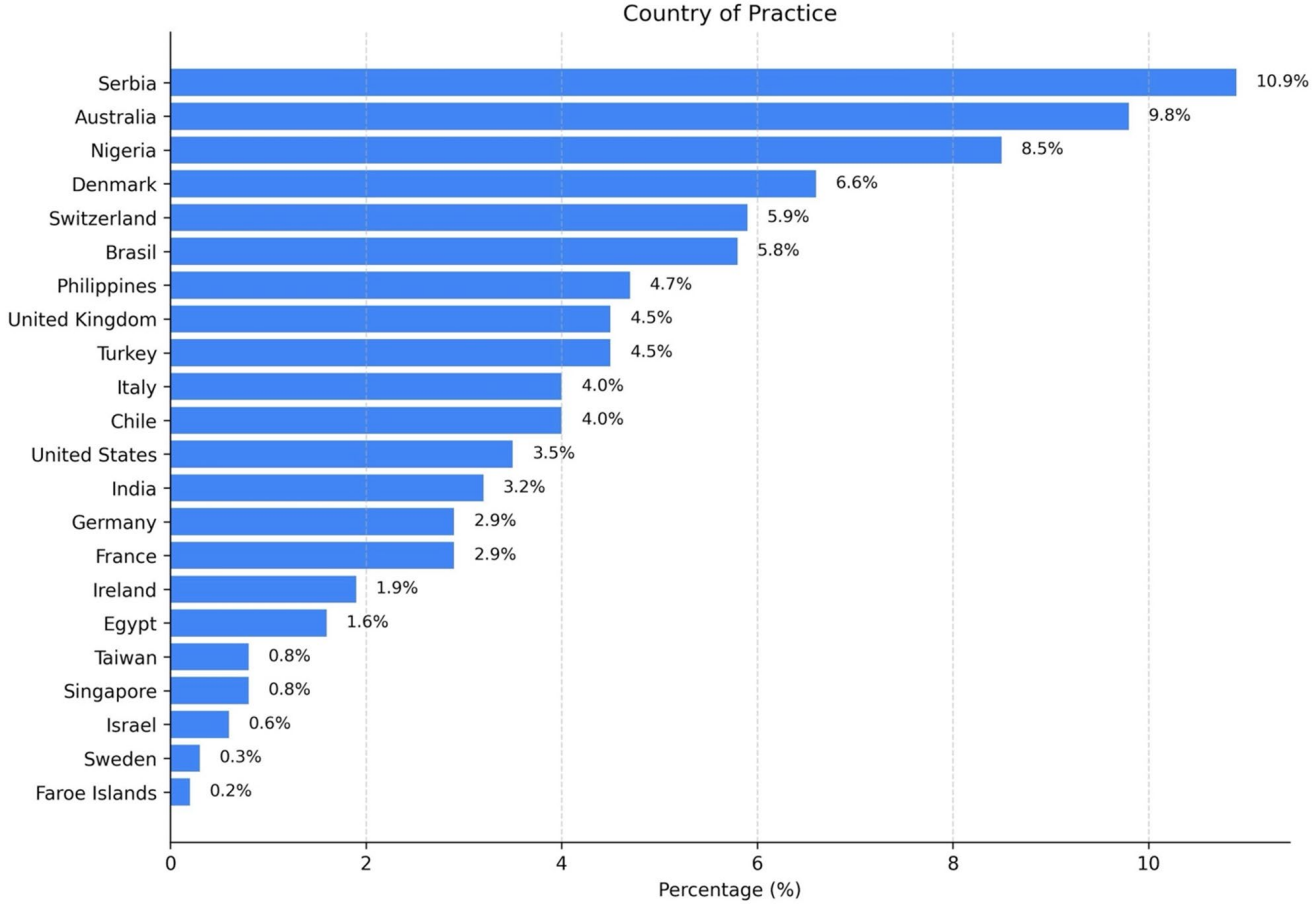
Geographic distribution of survey participants. Respondents represented 45 countries across six continents. The largest groups were from Serbia (10.9%), Australia (9.8%), Nigeria (8.5%), Denmark (6.6%), and Switzerland (5.9%); additional countries contributed smaller proportions

A total of 622 ophthalmologists from 45 countries, including Europe, South and North America, Africa, Asia and Oceania, participated in this global survey. The most represented countries were Serbia (10.9%), Australia (9.8%), Nigeria (8.5%), Denmark (6.6%), Switzerland (5.9%) and Brazil (5.8%). Additional notable contributions came from Germany, Turkey, the Philippines, the United Kingdom, Taiwan, Chile, Italy, India and the United States of America, each representing approximately 3% to 5% of respondents (Fig. 1).

**Fig. 2:**
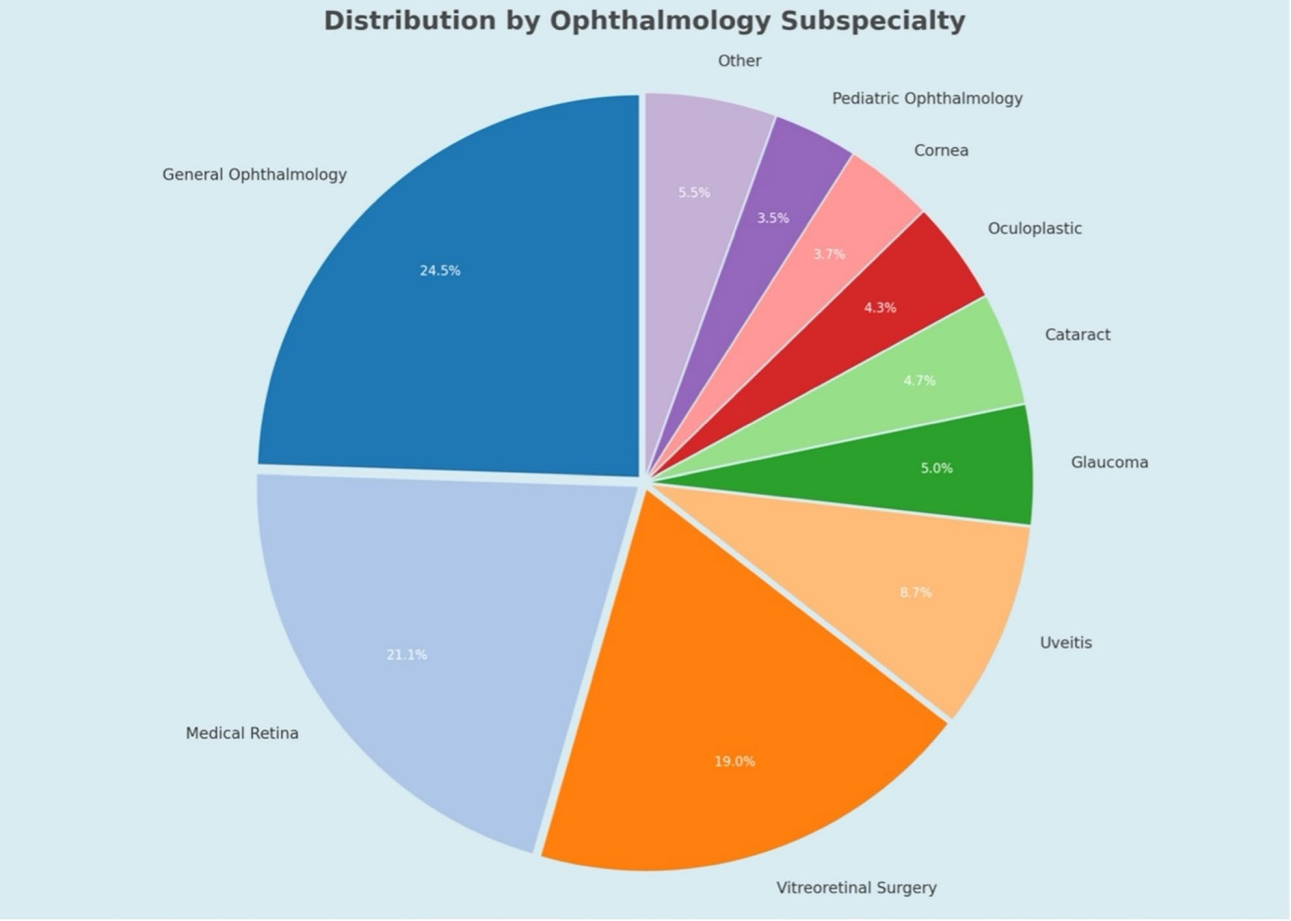
Distribution of ophthalmologists by subspecialty. The figure illustrates the distribution of survey participants according to their primary area of ophthalmology practice. The most represented subspecialties were general ophthalmology, medical retina, and vitreoretinal surgery. Other subspecialties included uveitis, glaucoma, cataract, oculoplastics, cornea, and pediatric ophthalmology

Regarding subspecialties, the majority were practicing in general ophthalmology (24.4%), medical retina (21.0%), or vitreoretinal surgery (18.9%). Other subspecialties included uveitis (8.7%), glaucoma (5.0%), cataract surgery (4.7%), oculoplastic surgery (4.3%), cornea (3.7%), pediatric ophthalmology (3.5%), neuro-ophthalmology (1.9%), ocular oncology (1.8%), and strabismus (1.8%) (Fig. 2).

In terms of clinical experience, 28.1% of the participants had been practicing for less than 5 years, 26.2% for 5–10 years, 24.9% for 11–20 years, and 20.7% for over 20 years.

Most respondents worked in medium-sized (6–20 practitioners, 34.4%) or large (21–50 practitioners, 27.0%) institutions. A majority (64.8%) reported affiliation with an academic or research institution.

### Knowledge and familiarity with AI

Approximately half of participants (48.2%) reported being somewhat familiar with AI, while 34.2% were not very familiar, 11.3% were very familiar, and 6.3% not familiar at all.

**Fig. 3:**
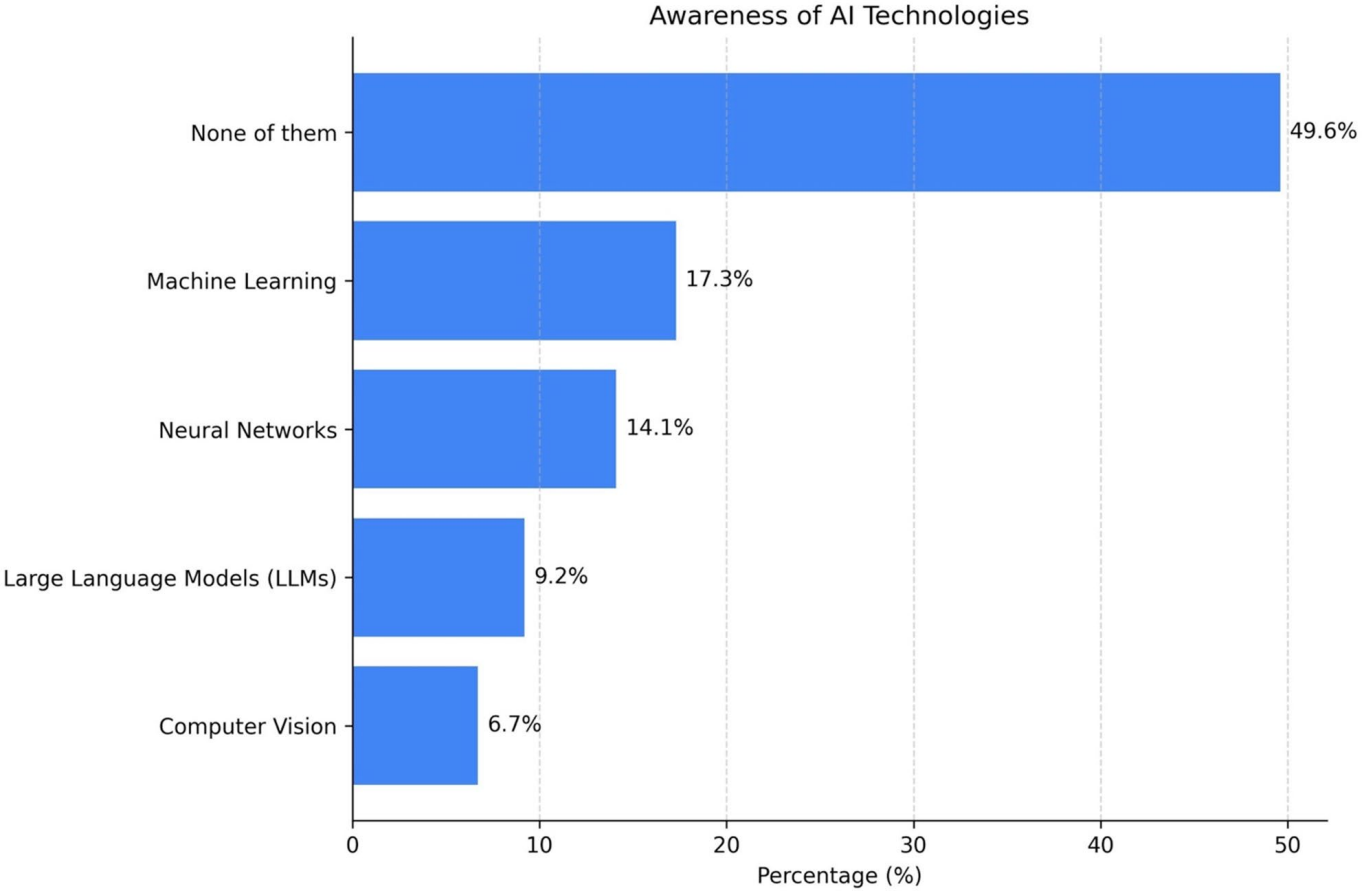
Awareness of artificial intelligence technologies among participants. Almost half of respondents reported no familiarity with any specific AI technology. Among those familiar with AI, machine learning and deep learning were the most frequently recognized fields, followed by neural networks, large language models, and, less commonly, computer vision

When asked about specific AI technologies, 49.6% were unfamiliar with any listed fields, while machine learning (17.3%) and deep learning (14.1%) were the most recognized technologies (Fig. 3).

Regarding confidence in understanding AI algorithm development and validation, 42.1% were not very confident, 27.3% somewhat confident, 24.6% not confident at all, and only 5.9% very confident. Ophthalmologists affiliated with academic institutions demonstrated significantly greater familiarity with AI compared to non-academic practitioners (*p* = 0.0011). No statistically significant associations were found between familiarity with AI and area of practice (*p* = 0.3497), years of practice (*p* = 0.3723), or size of the institution (*p* = 0.642).

### Adoption of AI in clinical practice

Among participants, only 7.2% reported regular use of AI tools in clinical practice, while 24.4% reported occasional use. The majority (52.6%) had not yet used AI but intended to in the future, while 15.8% did not plan to use AI.

Diagnostic tools (10.9%) and patient management systems (6.4%) were the most commonly used AI applications, while 73.4% reported no current use of any AI-based tool.

Use of AI tools was significantly more frequent among ophthalmologists affiliated with academic institutions, rather than non-academic ones (*p* = 0.034).

The main barriers to AI adoption included lack of knowledge or training (20.5%), cost of implementation (16.5%), concerns about reliability (12.9%), and lack of regulatory approval (10.3%).

### Perceptions of AI research and clinical impact

When assessing the perceived quality of current AI research in ophthalmology, 31.4% rated it as moderate, 25.1% as high, and 5.0% as very high; however, 33.4% indicated insufficient knowledge to make an evaluation. A strong positive association was observed between familiarity with AI and the perceived quality of AI research (*p* < 0.001).

Regarding the translation of AI research into clinical practice, 55.8% believed it was occurring to some extent, and 26.5% believed it was definitely occurring.

### Future expectations and concerns regarding AI

**Fig. 4:**
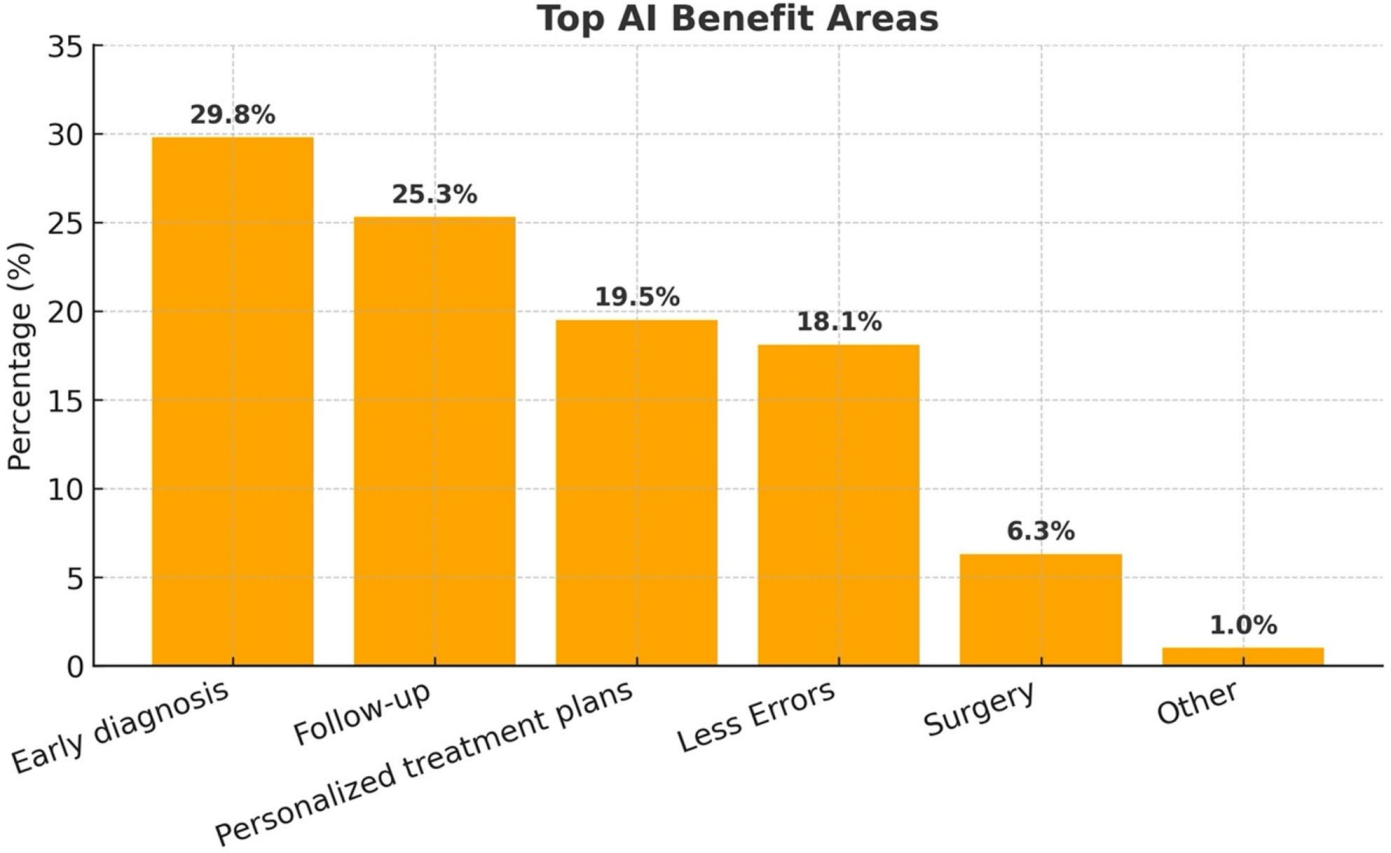
Areas of healthcare perceived to benefit the most from artificial intelligence. Distribution of ophthalmologists’ responses on the clinical domains where AI is expected to provide the greatest benefit. Early diagnosis, patient follow-up, and personalized treatment planning were the most frequently cited areas, followed by error reduction and surgical support

Most of the participants expressed high expectations for the impact of AI on patient outcomes, with 35.7% rating its potential as high and 33.8% as very high. The areas most expected to benefit from AI included early disease diagnosis (29.4%), patient follow-up and monitoring (25.4%), personalized treatment plans (19.6%), and reducing medical errors (18.5%) (Fig. 4).

Perception of AI’s potential was significantly associated with years of clinical experience (*p* = 0.0004), with more experienced ophthalmologists demonstrating a more positive outlook.

In terms of future roles, 53.2% believed that AI would complement but not replace traditional methods, while 37.6% anticipated AI becoming an essential tool in daily practice.

Major concerns about AI adoption included over-reliance on technology (26.1%), misinterpretation of AI recommendations (21.7%), loss of human touch (19.9%), and data privacy issues (16.9%).

### Ethical and legal considerations

Participants expressed moderate to strong concern about the ethical implications of AI in ophthalmology, with 33.4% reporting moderate concern, 24.3% slight concern, and 20.7% reporting that they were very concerned. Concerns about AI bias were widespread, with 44.2% believing bias could occur to some extent, and 18.3% believing bias would ‘definitely occur’. In this regard. Concerns about AI bias was higher among regular/occasional users than among non-users (*p* = 0.04).

Regarding liability, 40.2% believed that responsibility should be determined on a case-by-case basis, and 36.7% favored shared liability between AI developers and healthcare providers.

Confidence in AI’s ability to maintain patient confidentiality was moderate among most respondents (43.9%).

### AI training and educational needs

A large majority (75.1%) expressed willingness to undergo training to improve their understanding and use of AI, with 22.7% indicating conditional willingness depending on available time.

There was a strong association between willingness to undergo AI training and belief in the practical translation of AI research into clinical tools (*p* < 0.001).

**Fig. 5:**
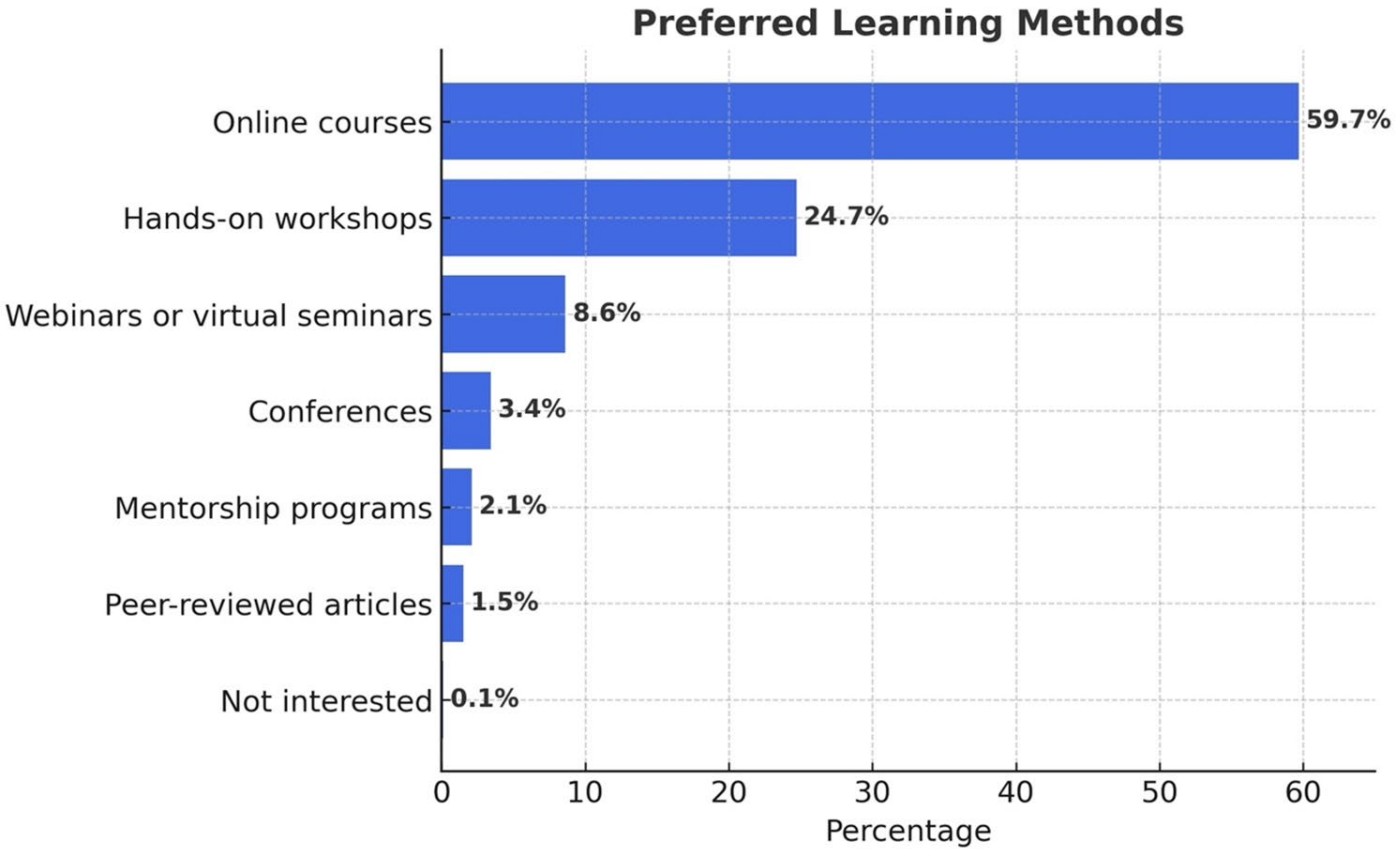
Preferred educational formats among respondents. Online courses were most preferred (59.7%), with hands-on workshops a distant second (24.7%); webinars, conferences, and mentorship programs were chosen far less often

Preferred educational methods included online courses (61.4%), hands-on workshops (25.4%), and webinars or virtual seminars (8.8%) (Fig. 5).

Regarding formal education, 46.1% supported offering AI-related courses as electives, while 39.5% favored integrating AI into the core ophthalmology curriculum.

### Regional subgroup analysis

No statistically significant differences were found in AI familiarity (*p* = 0.176), preferred educational formats (*p* = 0.807), willingness to undergo training (*p* = 0.296), current use of AI tools in clinical practice (*p* = 0.096), concern about bias (*p* = 0.053), or perceived potential to improve outcomes (*p* = 0.219) based on geographic location. However, a significant difference was observed in the overall level of ethical concern about AI (*p* = 0.018). Specifically, the proportion of respondents expressing high concern (score 4–5 on a 5-point scale) was highest in North America (50.0%), followed by South America (32.0%), Europe (29.1%), and Oceania (27.9%). In contrast, lower concern levels were found in Asia (13.9%) and Africa (8.0%).

## Discussion

This international survey provided a comprehensive overview of current attitudes, readiness, and barriers of eye doctors around the world to the next, unavoidable integration of AI in ophthalmology. Despite widespread openness and enthusiasm with 69.5% of respondents rating AI’s potential to improve clinical outcomes from moderate to very high, the actual adoption of AI in clinical practice remains limited, with only 7.2% reporting regular use, which is likely influenced by the fact that currently only a handful of licensed AI applications are available in ophthalmology [8, 18–20].

In particular for retinal care, near-term AI applications include OCT-based biomarker quantification and automated triage for diabetic retinopathy screening, as well as longitudinal monitoring in macular diseases (such as age-related macular degeneration and diabetic macular edema) [21–23]. In vitreoretinal surgery, AI can support case prioritization and surgical planning, and may enhance intraoperative imaging interpretation, helping to standardize decision-making.

In our survey, nearly half of respondents lacked familiarity with any specific AI technologies, especially those who were not part of academic institutions. This discrepancy may reflect a concept–practice gap: many clinicians recognize AI at a high level, but they lack technical familiarity with its subfields, which helps explain low adoption and underscores the need for skills-based training. However, despite this limited adoption, there is a clear willingness to engage with AI: over half of participants expressed intent to use AI in the future, and many recognized its value for early diagnosis, patient monitoring, and personalized care. Our findings highlighted critical current challenges to bridge this gap, namely the need for appropriate training for all the ophthalmologists and infrastructure to translate this readiness into real-world application. In particular, the 33.4% of respondents who reported insufficient knowledge should be interpreted cautiously, as this group likely represents both a limitation of current exposure and a key target for future educational initiatives.

An important finding of our study was the association between academic affiliation and AI familiarity, suggesting that institutional exposure significantly influenced awareness and subsequent AI adoption. This trend was also evident in the Chinese national survey by Yun et *al.*, where respondents from medical fields and with higher academic qualifications demonstrated greater understanding and willingness to engage in AI education [24]. In this perspective, recent studies have reported the interests and concerns among medical students and physicians regarding career planning in the context of AI’s growing influence [25, 26]. Another survey from the UK has underscored the importance of early exposure to AI during undergraduate education as a strategy to cultivate long-term engagement. Doctors interviewed in that study expressed the need to “plant the seed early,” suggesting that by the time clinical responsibilities dominate, mindsets may become more resistant to technological integration [27].

Furthermore, in our study we demonstrated that experience level positively correlated with a stronger belief in AI potential. In fact, ophthalmologists with over 20 years of practice showed greater openness to AI-supported decision-making. In contrast with our findings, in another Australian and New Zealand survey involving 632 local clinicians, early-career physician anticipated a more profound workforce impact, whereas more experienced practitioners were more skeptical about the real applicability of AI in clinical practice [28]. This difference may reflect variations in the respondent cohort, differences in questionnaire framing, or greater awareness of AI among ophthalmologists in the intervening years.

The generally optimistic attitudes toward AI identified in our study have also been echoed across multiple medical specialties, including radiology, pathology, and also ophthalmology [26, 29, 30]; however, more cautious views have been reported among general practitioners and psychiatrists, who tend to emphasize the importance of interpersonal interaction and clinical intuition, and therefore may see less relevance for AI in their domains [31, 32]. These divergences showed how both specialty and seniority level can influence expectations around AI, reinforcing the need for targeted strategies to build confidence and engagement across clinician groups.

Ophthalmologists should not only increase their use of AI tools but also understand how these systems are developed, validated, and implemented in real-world settings. As the ultimate decision-makers in patient care, clinicians must be able to appraise and select appropriate AI solutions. This knowledge is essential for safe, effective, and ethical integration into clinical workflows.

Importantly, across all surveyed cohorts, AI was perceived not as a rival, but more as an augmented assistant. This likely may reflect the fact that ophthalmology has long been a specialty accustomed to adopting new technologies [33]. Previous studies consistently identified early disease detection, individualized treatment planning, and reduction of repetitive tasks as key clinical benefits for clinicians, aligning with our own results [24]. We believe that these insights highlight the need to develop structured, accessible training programs that prepare ophthalmologists to safely and actively integrate AI into practice.

In this direction, demand for AI education was clear in our survey with 75.1% of respondents willing to undergo training. These results were in line with a previous survey administered to pediatric ophthalmologists, where 71% of them supported integration of AI into residency programs [34]. Despite this enthusiasm, institutional preparedness remains low. In the Australian/New Zealand cohort, only 13.8% of clinicians felt their specialist colleges were adequately equipped for AI integration [28]. Overall, respondents across the studies emphasized the need for structured curricula, clear safety guidelines, and robust ethical framework, which are currently lacking. There is an urgent necessity to standardize this educational transition.

Ethical and clinical concerns remain an important barrier to AI adoption in ophthalmology. In our study, over 60% of respondents expressed moderate-to-high concern about bias, data privacy, and the erosion of human connection in ophthalmic care. These apprehensions have already been expressed in recent surveys, which have highlighted fears that AI could undermine the physician-patient relationship, reduce professional autonomy, or even devalue the medical profession [35–37]. A large multicenter study among 3,000 medical students found that while most recognized AI’s potential to reduce errors and enhance care, many were worried about its impact on trust, confidentiality, and job security. Importantly, nearly 100% of the students expressed the need for targeted training in both the practical and ethical use of AI, indicating that teaching clinicians the proper skills to use AI responsibly was as crucial as the technology itself [36]. These findings reinforce the need for ethical frameworks, transparent governance, and proactive involvement of professional bodies in guiding AI integration.

Furthermore, while most variables did not differ significantly by geographic region, we observed notable variation in the level of ethical concern surrounding AI. Respondents from North America expressed the highest levels of concern, followed by South America and Europe, whereas lower concern was reported in Asia and Africa. These differences may reflect regional variations in regulatory frameworks, digital health maturity, or cultural perspectives on data privacy and automation [38]. This shows the importance of tailoring AI education and implementation strategies to local contexts and values.

Additionally, the rapidly evolving AI landscape poses challenges for both developers and clinicians, as many recent models are trained on natural images and remain unvalidated on ophthalmic dataset [39]. This domain gap slows progress toward robust, clinically ready tools and contributes to clinicians’ low confidence. Until algorithms are tailored to ophthalmology’s specific imaging and use cases, their day-to-day utility will remain limited.

These technological limitations, combined with the barriers highlighted in our survey, such as limited training opportunities, concerns about reliability and ethics, and the cost of implementation, show the need for coordinated action. For departments and clinics that may be uncertain about how to move forward, these findings may offer a useful starting point pointing the importance of a structured education, institutional planning, and clear strategies for safe adoption of AI in clinical practice.

Our study has limitations common to questionnaire-based research, including self-selection bias, under-representation of certain regions, the lack of adjustment for demographic features including age and education, and varied interpretations of what constitutes AI.

In conclusion, ophthalmologists worldwide acknowledge AI’s transformative potential but remain cautious about its implementation. To realize its benefits, current efforts must include equitable training, rigorous validation, and ethical integration. With these safeguards, AI can become a valuable tool, complementing, but not replacing, the human role in clinical care.

## Data Availability

The datasets generated and/or analyzed during the current study are not publicly available due to institutional data protection policies but are available from the corresponding author (RA) on reasonable request.
